# Correspondence: Reply to ‘Analytical flaws in a continental-scale forest soil microbial diversity study'

**DOI:** 10.1038/ncomms15583

**Published:** 2017-06-06

**Authors:** Jizhong Zhou, Ye Deng, Lina Shen, Chongqing Wen, Qingyun Yan, Daliang Ning, Yujia Qin, Kai Xue, Liyou Wu, Zhili He, James W. Voordeckers, Joy D. Van Nostrand, Vanessa Buzzard, Sean T. Michaletz, Brian J. Enquist, Michael D. Weiser, Michael Kaspari, Robert Waide, Yunfeng Yang, James H. Brown

**Affiliations:** 1 State Key Joint Laboratory of Environment Simulation and Pollution Control, School of Environment, Tsinghua University, Beijing 100084, China; 2 Institute for Environmental Genomics, Department of Microbiology and Plant Biology and School of Civil Engineering and Environmental Sciences, University of Oklahoma, Norman, Oklahoma 73019, USA; 3 Earth Science Division, Lawrence Berkeley National Laboratory, Berkeley, California 94270, USA; 4 CAS Key Laboratory for Environmental Biotechnology, Research Center for Eco-Environmental Science, Chinese Academy of Sciences, Beijing 100085, China; 5 Department of Ecology and Evolutionary Biology, University of Arizona, Tucson, Arizona 85721, USA; 6 The Santa Fe Institute, 1399 Hyde Park Rd, Santa Fe, New Mexico 87501, USA; 7 EEB Graduate Program, Department of Biology, University of Oklahoma, Norman, Oklahoma 73019, USA; 8 Smithsonian Tropical Research Institute, Balboa 0843-03092, Republic of Panama; 9 Department of Biology, University of New Mexico, Albuquerque, New Mexico 87131, USA

Nature Communications 8: Article number: 15583; DOI: 10.1038/ncomms15583 (2017); Published: 06 06 2017

We greatly appreciate Tedersoo's^1^ interest in our recent publication^2^ concerning temperature effects on continental scale diversity of microbial communities in forest soils^2^. The Correspondence raises several questions, primarily regarding the approaches used in our study. While we welcome this debate, we disagree with the claims regarding our study design, statistical analyses and interpretations.

The first concern raised in the correspondence is related to the sampling design. In contrast to most traditional microbial biogeographic studies, we sampled microbial communities at multiple spatial scales. Specifically, we focused on many replicates at a small scale, which resulted in a tradeoff for fewer samples at a large scale. This design is appropriate for addressing our research questions because soil environments are highly heterogeneous, which could mask the effects of environmental variables such as temperature on microbial distributions at continental scales. Increased sampling effort with many replicates within a site is the most effective way to mitigate the impacts of such heterogeneity on detecting patterns of microbial community diversity. We have now conducted a simulation analysis (Supplementary Table 1), which shows that large within-site sample sizes are necessary for capturing the trends between microbial diversity and temperature. Also, the experimental design with extensive sampling within a site allows us to appropriately assess the large within-site variation due to heterogeneity, which is also critical for revealing differences between microbial communities across sites. To further demonstrate this point, a similar simulation was performed using well-known published data on plant diversity and stability^3^. Diversity effects on stability could only be observed when the within-treatment variation was captured by more than 19 out of the 30 replicates (Supplementary Table 2). In addition, the range of temperatures (2.5–27 °C) of our six forest sites is comparable to many other continental surveys for plants, animals and phytoplankton, and should be broad enough to detect the general patterns of temperature effects on ecological communities. However, we do acknowledge that the number of sites is limited, and more sites within and/or across continents should be examined to evaluate whether our results are applicable to other forest ecosystems. Thus, we pointed out this potential caveat in the original paper^2^.

The correspondence^1^ also questions our regression analyses. In the original version of the study, we used univariate linear regression, multiple linear regression, nonlinear univariate regression and more complex models (original Supplementary Tables 7–9)^2^. We have now also conducted partial correlation analysis (Supplementary Table 3). All of these analyses indicated that temperature is generally a better predictor of microbial diversity than other environmental variables. We also performed univariate and multiple hierarchical linear model (HLM) analyses (Supplementary Tables 4 and 5), where site was used as grouping factor to account for our nested sampling design, and the degrees of freedom for the variable(s) and intercept were 4 and 120, respectively. Since heteroscedasticity and non-normality of residuals were found in many cases of our HLM analysis, variance weights were introduced to allow different variances for various sites, and the significance test was based on bootstrapping rather than parametric tests. The HLM analyses showed the same trends as univariate and multiple linear regression (original Table 9 and Supplementary Table 6, ref. 2), which further supports our original conclusion.

The third point is related to the use of Chao1 estimation. In this study, we did consider sequencing artefacts by removing singletons from raw sequencing data before resampling, which could improve sequence quality and thus subsequent analysis^4^. Also, our conclusions were supported by various diversity indexes showed the same pattern as Chao1, including Shannon, Inverse Simpson, Faith's phylogenetic diversity and the net relatedness index (original Supplementary Tables 6 and 7, ref. 2), as well as the Hill number^5^ (Supplementary Tables 6 and 7). Therefore, we do not believe the Chao1 estimation introduced significant bias in our conclusions.

Another critique is that this study did not cite all of the relevant literature. This was a result of space limitations, although we note that we were aware of all of this literature before publication of our original paper. Importantly, none of this literature provides explicit evidence that temperature is more important than other proposed environmental drivers in shaping microbial diversity, particularly in forest soils. Thus, this literature does not invalidate our original statement that ‘This is the first demonstration that temperature plays a more primary role in shaping variation in microbial diversity in the forest soils than other proposed environmental drivers.' (ref. 2).

In conclusion, we believe that (i) the approaches used in our study were appropriate for addressing our research questions of interest, (ii) the experimental results were cautiously interpreted and (iii) the conclusions were sound and solid.

## Additional information

**How to cite this article:** Zhou, J. *et al*. Reply to ‘Analytical flaws in a continental-scale forest soil microbial diversity study'. *Nat. Commun.* **8,** 15583 doi: 10.1038/ncomms15583 (2017).

**Publisher's note**: Springer Nature remains neutral with regard to jurisdictional claims in published maps and institutional affiliations.
